# Predicting ^18^F-FDG SUVs of metastatic pulmonary nodes from CT images in patients with differentiated thyroid cancer by using a convolutional neural network

**DOI:** 10.3389/fendo.2023.1127741

**Published:** 2023-05-04

**Authors:** Nianting Ju, Liangbing Nie, Yang Wang, Liying Hou, Chengfan Li, Xuehai Ding, Quanyong Luo, Chentian Shen

**Affiliations:** ^1^ Department of Nuclear Medicine, Shanghai Sixth People’s Hospital Affiliated to Shanghai Jiao Tong University School of Medicine, Shanghai, China; ^2^ School of Computer Engineering and Science, Shanghai University, Shanghai, China

**Keywords:** standard uptake value, lung metastases, differentiated thyroid cancer, prediction model, convolutional neural network

## Abstract

**Purpose:**

The aim of this study was to predict standard uptake values (SUVs) from computed tomography (CT) images of patients with lung metastases from differentiated thyroid cancer (DTC-LM).

**Methods:**

We proposed a novel SUVs prediction model using 18-layer Residual Network for generating SUVmax, SUVmean, SUVmin of metastatic pulmonary nodes from CT images of patients with DTC-LM. Nuclear medicine specialists outlined the metastatic pulmonary as primary set. The best model parameters were obtained after five-fold cross-validation on the training and validation set, further evaluated in independent test set. Mean absolute error (MAE), mean squared error (MSE), and mean relative error (MRE) were used to assess the performance of regression task. Specificity, sensitivity, F1 score, positive predictive value, negative predictive value and accuracy were used for classification task. The correlation between predicted and actual SUVs was analyzed.

**Results:**

A total of 3407 nodes from 74 patients with DTC-LM were collected in this study. On the independent test set, the average MAE, MSE and MRE was 0.3843, 1.0133, 0.3491 respectively, and the accuracy was 88.26%. Our proposed model achieved high metric scores (MAE=0.3843, MSE=1.0113, MRE=34.91%) compared with other backbones. The predicted SUVmax (R^2 =^ 0.8987), SUVmean (R^2 =^ 0.8346), SUVmin (R^2 =^ 0.7373) were all significantly correlated with actual SUVs.

**Conclusion:**

The novel approach proposed in this study provides new ideas for the application of predicting SUVs for metastatic pulmonary nodes in DTC patients.

## 1 Introduction

Thyroid cancer is the most common malignancy in the endocrine system (1). In China, it accounts for 4.7% of all cancer incidence and is expected to have 224,023 new patients in 2022 (2). Thyroid cancer is divided into subspecies of differentiated thyroid carcinoma (DTC), anaplastic thyroid carcinoma (ATC), and medullary thyroid carcinoma (MTC). DTC mainly includes papillary thyroid carcinoma (PTC), follicular thyroid carcinoma (FTC) and Hürthle cell carcinoma, which accounts for 94% of thyroid cancer and has a relatively good prognosis after standardized treatment (3, 4). However, it has been reported in the literature that 5%-25% of DTC patients can develop distant metastasis (5, 6), with lung metastases being the most common, accounting for 55%-85% of cases (7–10). In addition, respiratory failure due to pulmonary metastases may be the leading cause of death in patients with lung metastases from differentiated thyroid cancer (DTC-LM), with approximately 50% of patients dying within 10 years (11, 12).

Based on iodine uptake capacity (9), lung metastases from DTC can be classified as ^131^I-avid and non ^131^I-avid (13). 18-fluorodeoxyglucose positron emission tomography/computed tomography (^18^F-FDG PET/CT) is mainly considered for high-risk DTC-LM patients with elevated thyroglobulin (Tg) and negative ^131^I whole-body scan (14). It can not only detect lung metastases with high sensitivity and specificity, but also predict the potential poor outcome of ^131^I therapy in ^18^F-FDG positive lesions (15, 16).

^18^F-FDG PET/CT is becoming more commonly used in clinical practice. However, the total cost of this examination ranged from 7,000 to 10,000 RMB, which hampers the implementation of PET/CT units and imposes a heavy financial burden on patients (17). According to the World Health Organization’s Global Atlas of Medical Devices, only 3% of upper-middle income, and 4% of lower-middle income countries possesses at least one PET scanner per million people. Furthermore, 95% of low income countries and 92% of lower-middle income countries don’t have an available PET/CT unit (18). Compared to PET/CT, CT scans are much more prevalent, especially in some developing countries. In China, the number of PET/CT was only 0.3 units per million people, which is much less than the 18 CT units per million people. To sum up, CT is a widespread and cost-effective alternative that can be used as a routinely used technique to analyze pulmonary nodules.

Artificial intelligence (AI) algorithms, especially deep learning, have an excellent performance in medical image analysis because of its ability to integrate vast datasets. Convolutional neural network (CNN) is one of the representative algorithms of deep learning. It has attracted more attention in radiological image processing with its powerful deep structure representation capability. CNN trained on millions of photographic images can be applied to medical images through transfer learning (19). The development of AI can be applied clinically to improve patient care by providing accurate and efficient decision support (20, 21).

In this study, we proposed a model with 18-layer Residual Network (ResNet-18) based on CNN to predict standard uptake values (SUVs) of pulmonary metastatic nodules in patients with DTC-LM from CT images. The proposed model can extract features of lung metastases automatically and predict the SUVmax, SUVmean and SUVmin based on CT images. Applying AI methods to CT images of patients with DTC-LM to achieve SUV prediction has great clinical implications.

## 2 Methods

### 2.1 Datasets

The data used in this study were retrospectively derived from the Department of Nuclear Medicine, Shanghai Sixth People’s Hospital from November 2014 to September 2021. The patients who satisfied the following criteria were finally enrolled: (1) differentiated thyroid carcer confirmed by pathological results after total or near total thyroidectomy; (2) all patients included had underwent ^18^F-FDG PET/CT scan; (3) more than three pulmonary nodes detected by ^18^F-FDG PET/CT and no suspected other primary tumor was found; (4) thyroid stimulating hormone (TSH)-suppressed Tg >1 ng/ml or TSH-stimulated Tg >10 ng/ml, or Tg antibody (TgAb) >100 IU/ml; (5) metastatic pulmonary nodes were confirmed on ^131^I-SPECT/CT scan after radioiodine therapy; (6) or pathologically proved metastatic pulmonary nodes from DTC. Exclusion criteria were as following: (1) with history of other malignancies; (2) other primary tumor confirmed by PET/CT images; (3) low-quality images; (4) loss of follow-up and unnecessary information.

^18^F-FDG PET/CT images were acquired according to standardized scanning protocols at our institution. All patients fasted and underwent PET/CT scans 60 minutes after receiving intravenous injection of a dose of 3.7 MBq ^18^F-FDG per kilogram of body weight. PET/CT equipment manufactured by GE Healthcare was used to generate the images. CT scanning was first performed for attenuation correction and anatomical localization, followed by PET emission scan to show ^18^F-FDG uptake, and PET/CT fusion images were displayed after processing. Nuclear medicine physicians measure SUV from the images at the workstation. This study was approved by the ethics committee of Shanghai Sixth People’s Hospital.

### 2.2 Network architecture

**Figure 1:**
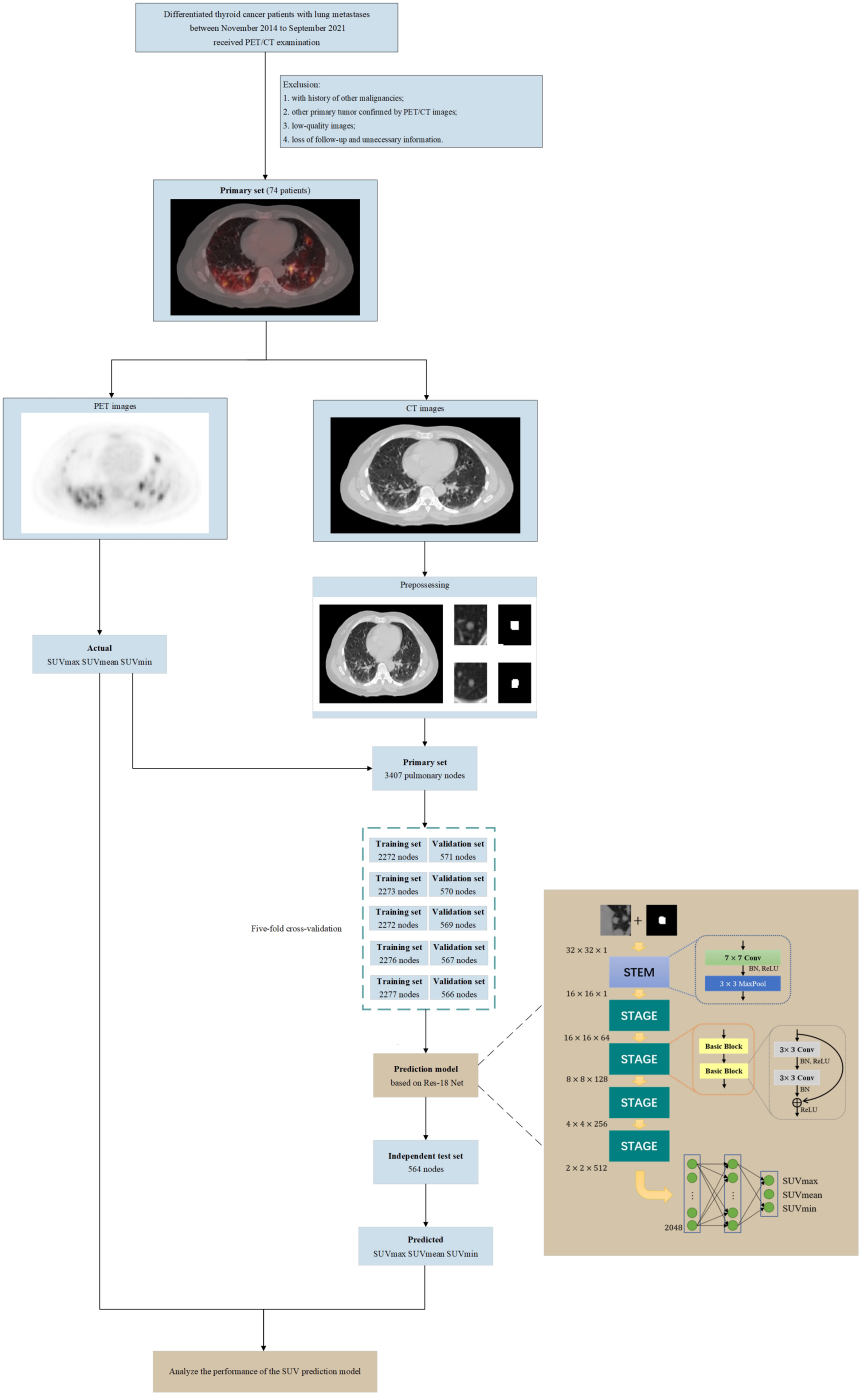
Flowchart of our study (including patient selection and exclusion criteria, image pre-processing, architecture of the SUV prediction model and model training, validation, testing).

To predict SUVs from CT images, the following points have to be considered: (1) DTC-LM patient’s metastatic pulmonary nodules should first be manually outlined by the specialists in the department of nuclear medicine as the regions of interest (ROI). (2) The original input CT image should be separated from the PET/CT images, which means does not contain PET images. (3) The CT images are grayscale images, which contain less information. The information of focused features such as pixel values need to be extracted to predict SUVs. Therefore, it is important to consider pixel values in the preprocess, the network structure, and loss function. Based on the above considerations, we designed this framework as in **Figure 1** to implement the prediction of SUVs from CT images.

### 2.3 Image preprocessing

The PET/CT images of the patients included in this study were stored in DICOM format. The CT image matrix was 512×512 with a thickness of 1.25 mm, and the PET image matrix was 128×128 with a thickness of 3.27 mm. We used Algorithm 1 to preprocess the CT images in DICOM format. We also converted the pixel values of PET images in DICOM format to SUVs according to the calculation method provided by GE health care. The PET images were resampled using the SimpleITK tool to make the spatial resolution consistent with the CT images.

Algorithm 1 Normalize based window center and width.
**Input:** DICOM file for CT 
$D_{dcm}$
Window center *W_c_
* and window width *W_w_
*
**Output:** 
$D_{out}$

**1.** read *Pixel Array, Rescale Slope* and *Rescale Intercept* from 
$D_{dcm}$

**2.** 
HounsfieldUnit=PixelArray×RescaleSlope+Rescale Intercept;

**3.** 
$top=W_{c}+W_{w}*0.5;$

**4.** 
$bottom=W_{c}-W_{w}*0.5;$
;
**5. for each** *hu* in *Hounsfield Unit* **do 6.** 
$hu=\{\begin{array}{c} top\;\;\;\;if\;hu\geq top\\ bottom\;\;\;\;if\;hu\leq bottom;\\ hu\;\;\;\;otherwise \end{array}$

**7.**

hu=hu÷(top−bottom);

**8. end 9. return** *Hounsfield Unit;*

To protect patients’ privacy and uniform the image size, we first removed all the information related to the patient from the image includes age, name and patient number. The ROI of each lung nodule were then created manually by a specialist in the department of nuclear medicine. Finally, we obtained images of 3407 lung nodules of different sizes. We preprocessed each image of labeled lung nodules as shown in **Supplementary Figure 1**. The purpose of this was to remove irrelevant areas and interference markers to prevent the adverse effect of noise on performance. The labeled lung nodule was first positioned and then cropped it into an image of 32×32 pixels centered on the lung nodule. If the labeled lung nodule was larger than 32×32 pixels, a square was cropped out with the longest edge of the lung nodule region and resize to 32×32 pixels. If there were multiple pulmonary nodules in the cropped slice, we first found the labeled pulmonary nodule by the algorithm, used it as an image center and cropped it a pixel of 32×32 . Then we set the pixel value to one for the labeled pulmonary nodes in the image, while the other regions outside the labeled pulmonary nodes were filled with pixel value zero. Finally, an image of 32×32 pixels containing a single pulmonary nodule was input into the regression prediction part of the model for predicting SUVs.

### 2.4 Model structure

In this study, we used 34-layer Residual Network (ResNet-34) as the backbone part of the network, which composed of one 7×7 convolutional layer, eight basic blocks, one max pooling layers, and two fully connected layers to realize the automatic prediction of SUVs on CT images. The STEM contains a 7×7 convolutional layer, and a max pooling layer. It is worth noting that we have made minor changes to the STEM part of the model. The input to the model was the normalized CT data with the labeled data, calculated as follows:

$input=\left(D_{out}+mask\right)*0.5$

Where, $D_{out}$ represents the normalized CT image, mask is the labeled data.

Each STAGE contained two basic blocks. Unlike conventional CNN stacked by multiple convolutional layers and pooling layers, each basic block was composed of two 3×3 convolutional layers and a short connection. In the basic block, activation function uses ReLU. Shortcut connections can make the deep network easier to optimize and solve the degradation problem caused by deep networks. Finally, there were two fully connected layers that collect and classify the extracted features. The first fully connected layer was followed by the Gaussian Error Linear Unit (GeLU) function and the Dropout layer, and the second fully connected layer was followed by the Dropout layer. The dimensions of feature maps after each layer were shown in **Supplementary Table 1**.

### 2.5 Experimental settings and evaluation indicators

We randomly split the dataset into two subsets, a training and validation set containing 2843 nodes and an independent test set containing 564 nodes. For the training, we used L1 Loss function and the standard stochastic gradient descent (SGD) optimizer with a momentum of 0.8, and a weight decay of 0.0005. The batch size was set to 32, and the dropout rate was set to 0. The training epoch was set to 200. The learning rate will be multiplied by 0.1 at the half and three-quarters of the training epoch.

To evaluate the prediction effect of each model objectively and select the final model, we performed five-fold cross-validation of the model. The training and validation set was divided into five nonoverlapping subdatasets randomly. Then the model was trained and validated five times. Four subdatasets were used to train the model, and the remaining one subset is used to validate the model’s performance. Moreover, each model needed to be trained and validated five times, and the independent test set was also used to test the model’s performance.

Besides, we applied multiple evaluation indicators to estimate the performance of the model. The performance of the regression task was quantified by three metrics: mean absolute error (MAE), mean squared error (MSE), and mean relative error (MRE).

MAE measured the average absolute error between the predicted value and the actual SUVs on the dataset, while MRE measured the average relative error. MSE reflected the absolute deviation of the predicted value from the actual SUVs. They were defined as follows:

MAE


$$=\frac{\sum_{i=1}^{n}\left|predicted_{i}-actual_{i}\right|}{n}$$

MSE=


$$\sqrt{\frac{\sum_{i=1}^{n}{\left(predicted_{i}-actual_{i}\right)}^{2}}{n}}$$

MRE=


$$\frac{\sum_{i=1}^{n}\frac{\left|predicted_{i}-actual_{i}\right|}{predicted_{i}}}{n}$$

In these equations above, n represents the number of images (equal to 3407 in our experiment), *predicted_i_*, represents the predicted SUVs of i-th nodes, while *actual_i_* represents the actual SUVs.

To further measure the performance of the model, we classified the SUV according to different intervals, using classification metrics to measure. The performance of the classification task was evaluated using standard metrics including specificity, sensitivity, F1 score, positive predictive value (PPV), negative predictive value (NPV) and accuracy. They derived from true-positive (TP), true-negative (TN), false-positive (FP), and false-negative (FN) as follows:


Specificity= TN/(TN+FP)

Sensitivity= TP/(TP+FN)

F1 score= (2×PPV×Sensitivity)/(PPV+Sensitivity)

PPV= TP/(TP+FP)

NPV= TN/(TN+FN)

Accuracy= (Sensitivity+Specificity)/2

### 2.6 Statistical analysis

The DICOM files of PET/CT were preprocessed using Python toolkits such as Numpy 1.22.3, SimpleITK 2.1.1.1, OpenCV-python 4.6.0.66 and Pydicom 2.3.0. The deep learning network was implemented in Python using PyTorch 1.8.0. The deep learning models were trained, validated and tested on a server with an NVIDIA Tesla V100 PCIe 32GB and an Intel Xeon Gold 5115 CPU. Correlation analysis was performed in the statistical software GraphPad Prism 9.

## 3 Results

### 3.1 Patient and image characteristics

**Figure 2:**
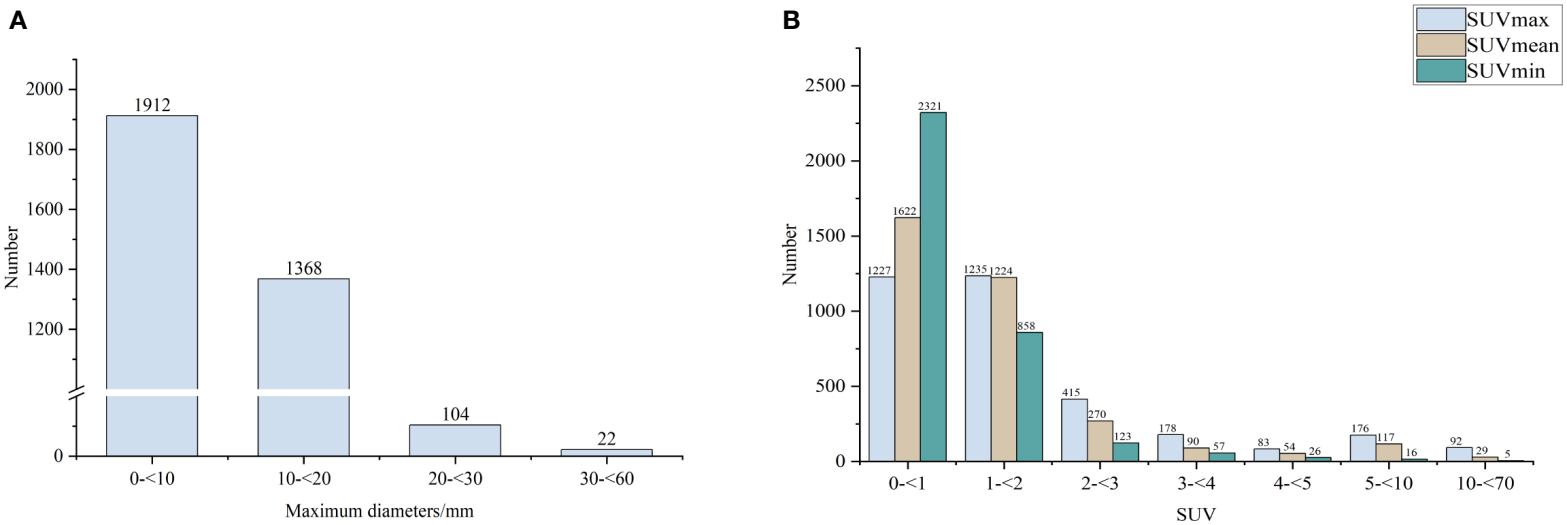
The distribution of maximum diameters **(A)** and SUV **(B)** of the nodes.

The number of patients analyzed in the study was 74, including 46 males and 28 females, The youngest of them was 11 years old and the oldest was 82, with a mean age of 55.2 years. The clinical characteristics of the patients were shown in **Table 1**. The collected dataset contained 3407 PET/CT images of pulmonary nodules correctly marked by the nuclear medicine specialists. Each contained an 32×32 CT image and an 32×32 annotated image. The median maximum diameter of these nodules was 9.77mm and the median actual SUVmax, SUVmean, SUVmin measured by PET images were 1.31, 1.06, 0.81, respectively. Their distribution was shown in **Figure 2**.

**Table 1:** Patients’ clinical characteristics.

Characteristics	Total (n=74)
Gender	
Males	28 (37.8%)
Females	46 (62.2%)
Age (years)	57 (45,66)
Pathological types	
PTC	56 (75.7%)
FTC	15 (20.3%)
PDTC	3 (4.1%)
Tg level^*^ (ng/ml)	147.60 (23.03,815.00)
TgAb level^#^ (IU/ml)	11.80 (10.54,18.68)
TSH level^**^ (mU/l)	0.10 (0.02,2.44)
Maximum Diameter (mm)	9.77 (7.87,12.49)
Actual SUVmax	1.31 (0.87,2.20)
Actual SUVmax	1.31 (0.87,2.20)
Actual SUVmean	1.06 (0.75,1.63)
Actual SUVmin	0.81 (0.59,1.14)

Values are presented as number (percentage) or median (Q25, Q75).

PTC, papillary thyroid carcinoma; FTC, follicular thyroid carcinoma; PDTC, poor differentiated thyroid carcinoma; Tg, thyroglobulin; TgAb, thyroglobulin antibody; TSH, thyroid stimulating hormone; SUV, standard uptake value.

^*^Tg <0.040 was calculated as 0.04 ng/ml, Tg >25000 ng/ml was calculated as 25000 ng/ml

^#^TgAb<10 IU/ml was calculated as 10 IU/ml, TgAb>4000 UI/ml was calculated as 4000 IU/ml.

^**^TSH<0.005 mU/l was calculated as 0.005 mU/l.

### 3.2 Performance in regression task

The performance of our proposed model in regression task was shown in **Table 2**. The average MAE, MSE and MRE of SUVmax, SUVmean and SUVmin on five-fold cross-validation set was 0.3493, 1.0880 and 0.3236 respectively. The average MAE, MSE and MRE of SUVmax, SUVmean and SUVmin on independent test set was 0.3843, 1.0133 and 0.3491 respectively.

**Table 2:** Performance of the SUV prediction model in the regression task.

Metric	Five-fold cross-validation				Independent test			
	SUVmax	SUVmean	SUVmin	AVG	SUVmax	SUVmean	SUVmin	AVG
MAE	0.5344 (0.4839, 0.5889)	0.3240 (0.2958, 0.3539)	0.1895 (0.1759, 0.2039)	0.3493 (0.3194, 0.3810)	0.5894 (0.5403, 0.6416)	0.3536 (0.3279, 0.3806)	0.2100 (0.1972, 0.2234)	0.3843 (0.3563, 0.4139)
MSE	2.3494 (1.5690, 3.3032)	0.7323 (0.5315, 0.9684)	0.1821 (0.1397, 0.2301)	1.0880 (0.7551, 1.4902)	2.2289 (1.6079, 2.9677)	0.6341 (0.4998, 0.7852)	0.1708 (0.1400, 0.2047)	1.0133 (0.7576, 1.3081)
MRE	36.41% (27.09%, 51.97%)	31.89% (23.22%, 46.26%)	28.76% (20.91%, 41.78%)	32.36% (23.80%, 46.64%)	38.37% (33.12%, 44.36%)	34.19% (29.04%, 40.10%)	32.17% (27.11%, 38.03%)	34.91% (29.81%, 40.75%)

Results are presented as values (95% Confidence Interval).

SUV, standard uptake value; AVG, average; MAE, mean absolute error; MSE, mean square error; MRE, mean relative error.

### 3.3 Correlation between the predicted SUVs with actual SUVs

**Figure 3:**
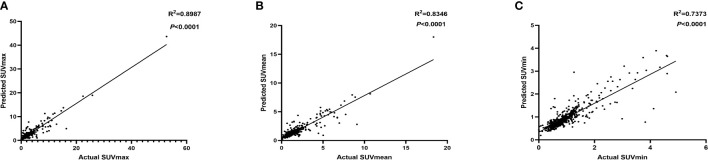
The correlation between the predicted SUVmax **(A)**, SUVmean **(B)**, SUVmin **(C)** with actual SUV in independent test set.

We evaluated the correlation between the predicted SUVs with actual SUVs of each node in the independent test set (**Figure 3**). SUVmax (R^2 =^ 0.8987, *P*<0.001), SUVmean (R^2 =^ 0.8346, *P*<0.001), SUVmin (R^2 =^ 0.7373, *P*<0.001) were found to be significantly and positively correlated for all of them.

### 3.4 Performance in classification task

The performance of our proposed model in classification task was shown in **Table 3**. The classification task was calculated after we perform coarse classification according to four intervals based on the SUVmax predicted by the model and the real SUVmax. In the classification task of dividing the actual value of SUVmax into four intervals [0,2.5) ,  [2.5,5) , [5,10) and [10,+∞) , the accuracy is 89.87% on the five-fold cross-validation set and 88.26% on the independent test set.

**Table 3:** Performance of the SUV prediction model in the classification task.

Metrics	Five-fold cross-validation					Independent test			
	[0,2.5)	[2.5,5)	[5,10)	[10, + ∞		[0,2.5)	[2.5,5)	[5,10)	[10, + ∞
Spec (%)	76.38	95.90	98.44	99.75		71.38	95.41	98.39	99.67
Sen (%)	96.42	65.99	59.89	65.50		95.94	58.89	57.93	58.67
F1 (%)	95.24	67.73	63.52	74.64		94.36	61.86	61.78	68.40
PPV (%)	94.10	70.18	67.81	88.65		92.84	65.20	66.54	83.70
NPV (%)	84.73	95.18	97.83	99.03		81.97	94.07	97.74	98.88
Acc (%)	89.87 (88.74, 90.96)					88.26 (87.06, 89.43)			

Accuracy is presented as values (95% Confidence Interval).

Spec, specificity; Sen, sensitivity; PPV, positive predictive value; NPV, negative predictive value; Acc, accuracy.

### 3.5 Models with different backbones

We compared the proposed model with convolutional neural networks using ResNet, DenseNet, EfficientNet and ConvNexXt as backbones on the five-fold cross-validation set and independent test set. The results can be seen in **Table 4**. The MAE, MSE and MRE in independent test is 0.3843, 1.0113 and 34.91% respectively. Our proposed model with ResNet18 as the backbone performs better compared with other backbones when considering these three metrics together.

**Table 4:** Comparison of regression performance with other backbones.

Backbone	Five-fold cross-validation			Independent test		
	MAE	MSE	MRE	MAE	MSE	MRE
ResNet18	0.3914	1.2939	33.89%	0.4201	1.2103	39.84%
ResNet34	0.3702	1.0966	33.12%	0.4051	1.1168	36.49%
ResNet50	0.3918	1.4110	34.66%	0.4182	1.1792	37.83%
DenseNet121	0.4188	1.3539	36.20%	0.4778	1.7737	38.99%
DenseNet169	0.4071	1.2794	34.73%	0.4792	1.8015	39.87%
DenseNet201	0.4266	1.4281	35.64%	0.4798	1.6640	41.47%
EfficientNet-B0	0.5449	2.2230	41.38%	0.6038	3.2052	45.26%
EfficientNet-B1	0.5175	2.1594	51.09%	0.5828	2.7543	45.40%
EfficientNet-B2	0.5250	2.4541	39.16%	0.5750	2.6721	43.99%
ConvNeXt-T	0.5883	2.4648	64.47%	0.6319	2.6453	50.72%
ConvNeXt-S	0.5854	2.4439	63.46%	0.6375	2.4818	51.25%
ConvNeXt-B	0.5760	2.4626	60.20%	0.6325	2.6616	51.48%
Proposed model	0.3493	1.0880	32.36%	0.3843	1.0113	34.91%

MAE, mean absolute error, MSE: mean squared error; MRE, mean relative error.

### 3.6 Influence factors of SUVs prediction

We analyzed the correlation between the maximum diameter and actual SUVs with MAE, MRE and MSE in the independent test set (**Table 5**). The correlation coefficients were 0.190, 0.612, 0.667, 0.557 for MAE, and 0.162, 0.612, 0.610, 0.426 for MSE respectively, all of which were statistically significant. The correlation coefficients for MRE were not statistically significant.

**Table 5:** Correlation analysis of errors.

Correlation coefficient	MAE	MRE	MSE
Maximum diameter	0.190^***^	-0.041	0.162^***^
Actual SUVmax	0.612^***^	-0.046	0.612^***^
Actual SUVmean	0.667^***^	-0.074	0.610^***^
Actual SUVmin	0.557^***^	-0.118^**^	0.426^***^

***P<0.001, **P<0.005.

MAE, mean absolute error; MRE, mean relative error; MSE, mean squared error; SUV, standard uptake value.

## 4 Discussion

In this study, we collected data from 74 patients with DTC-LM and obtained PET/CT images of their 3407 pulmonary metastatic nodules. And, we proposed a model used ResNet-18 as backbone to predict SUVs on CT images for the first time. This CNN architecture was trained and validated using 2843 CT images and tested using 564 images. The experiment showed that our model could predict SUVs effectively. To evaluate the prediction effect of each model objectively, we used five-fold cross-validation and comparative analysis with other mainstream CNN models under three evaluation indicators, including MAE, MSE and MRE. The comparative experiment showed that the performance of model using ResNet-18 as backbone in predicting SUVs from CT images was better than other CNN models.

As one of the most curable cancers, DTC has a favorable prognosis carrying a 10-year overall survival rate of about 90% (22). Although distant metastasis is not a frequent event in DTC, it has adverse impact on survival (23). Specially, DTC-LM patients with ^18^F-FDG positive/^131^I negative pulmonary metastases may have shorter survival due to their insensitivity to radioiodine therapy. SUV is the most commonly used semiquantitative tool to measure FDG uptake. It not only reflects the absolute FDG uptake in the tumor, but also assess metabolic changes. Therefore, it is crucial to identity the level of pulmonary metastases FDG uptake in DTC-LM patients to make a therapeutic decision.

Compared to CT and MRI, PET/CT scan may be subject to equipment inaccessibility and the high cost for patients. This scan also requires the injection of ^18^F-FDG into the body which has a potential radiation risk to the operators. In addition, the distribution and uptake of ^18^F-FDG could be affected by blood glucose level, making it necessary to control the patient’s blood glucose level prior to the scan. These disadvantages hinder its routine implementation into medical field.

Currently, there are many studies on CT-based image recognition and prediction. Wang et al. developed a deep learning model using CT scans efficiently predicted EGFR mutational status in patients with NSCLC (24). Liu et al. developed a CT-based radiomic signature to predict the expression status of the genes encoding E- cadherin, Ki-67, VEGFR2 and EGFR, in patients with gastric cancer (25). To the best of our knowledge, predicting SUVs based on CT images has not been systematically reported. Our study, for the first time, proposed a logical and easy-to-use method to try to predict SUVs of pulmonary metastasis from thyroid cancer by using CT images.

There are still shortcomings in our study. Firstly, the number of lung nodules with large diameters and high SUVs is small, which may affect the generalization ability of this prediction model. Secondly, since only patients with thyroid cancer were included in the current study, the applicability of this model to pulmonary nodes from other malignancies still needs further validation in a larger dataset. In the future, we will continue to collect PET/CT images of metastatic pulmonary nodes and explore SUV prediction model with a better performance. Thirdly, it’s hard to explain why morphological features can predict molecular metabolism information. However, metabolic changes of tumor cells may affect their biological behavior and thus could finally alter anatomical imaging findings. So, it is possible that anatomical and metabolic information could be related.

In conclusion, we proposed a model to predict ^18^F-FDG SUVs of metastatic pulmonary nodes from CT images in patients with differentiated thyroid cancer by using a convolutional neural network for the first time. The novel model proposed in this study provides new ideas for applying artificial intelligence approaches to predict molecular metabolism information from anatomical features and may show good application potential in clinic.

## Data availability statement

The original contributions presented in the study are included in the article/**Supplementary Material**. Further inquiries can be directed to the corresponding authors.

## Author contributions

CS and XD contributed to conception of the study. YW and LH organized the database. LN and CL designed the model. NJ wrote the first draft of the manuscript. QL and CS revised the manuscript. All authors contributed to the article and approved the submitted version.

## Supplementary material

Supplementary Figure 1 The schematic diagram of the image pre-processing.

Supplementary Table 2 Dimensions of feature maps after each layer.
