# Supplementary figures and images for: Predicting 18F-FDG SUVs of metastatic pulmonary nodes from CT images in patients with differentiated thyroid cancer by using a convolutional neural network
Source: Front Endocrinol (Lausanne). 2023 May 4;14:1127741. doi: 10.3389/fendo.2023.1127741 (PMC10194030; doi:10.3389/fendo.2023.1127741)

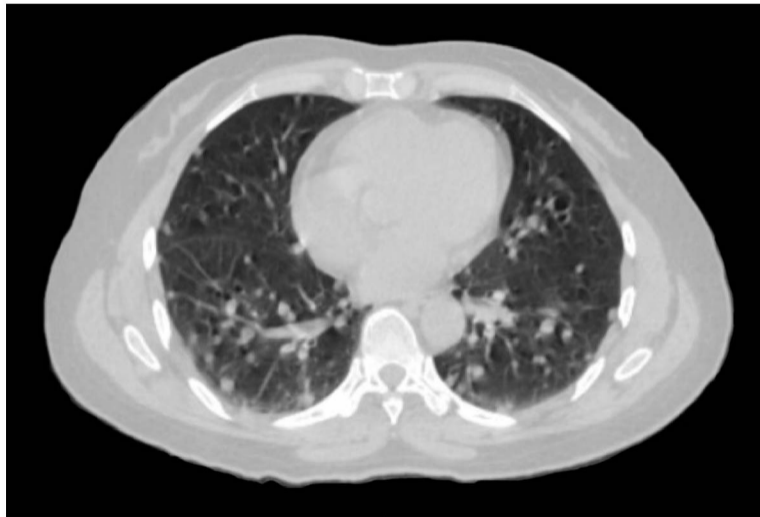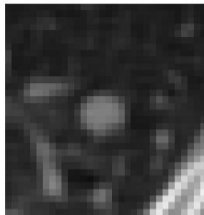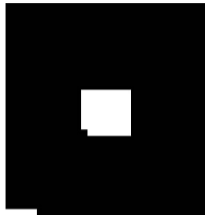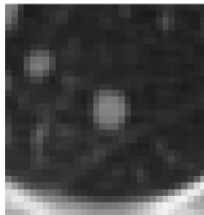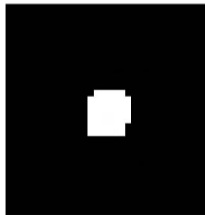

Supplement: Supplementary Figure 1 — The schematic diagram of the image pre-processing. [file DataSheet_1.pdf]
