# Supplementary material for: Predicting 18F-FDG SUVs of metastatic pulmonary nodes from CT images in patients with differentiated thyroid cancer by using a convolutional neural network
Source: Front Endocrinol (Lausanne). 2023 May 4;14:1127741. doi: 10.3389/fendo.2023.1127741 (PMC10194030; doi:10.3389/fendo.2023.1127741)
Supplement: Supplementary Table 2 — Dimensions of feature maps after each layer. [file DataSheet_2.docx]

Table 1 Dimensions of feature maps after each layer.

| Block | Layers | Output size |
| --- | --- | --- |
| Input | Input layer | 32×32×1 |
| STEM | 7×7 Conv+BN+ReLU | 16×16×1 |
|  | 3×3 max pooling |  |
| STAGE1 | 3×3 Conv+BN+ReLU | 16×16×64 |
|  | 3×3 Conv+BN |  |
|  | 3×3 Conv+BN+ReLU |  |
|  | 3×3 Conv+BN |  |
| STAGE2 | 3×3 conv | 8×8×128 |
|  | 3×3 conv |  |
|  | 3×3 conv |  |
|  | 3×3 conv |  |
| STAGE3 | 3×3 conv | 4×4×256 |
|  | 3×3 conv |  |
|  | 3×3 conv |  |
|  | 3×3 conv |  |
| STAGE4 | 3×3 conv | 2×2×512 |
|  | 3×3 conv |  |
|  | 3×3 conv |  |
|  | 3×3 conv |  |

Abbreviations: Conv: convolution; BN: Batch Normalization; ReLU: Rectified Linear Unit.
